# p120-Catenin Is Critical for the Development of Invasive Lobular Carcinoma in Mice

**DOI:** 10.1007/s10911-016-9358-3

**Published:** 2016-07-13

**Authors:** Milou Tenhagen, Sjoerd Klarenbeek, Tanya M. Braumuller, Ilse Hofmann, Petra van der Groep, Natalie ter Hoeve, Elsken van der Wall, Jos Jonkers, Patrick W. B. Derksen

**Affiliations:** 1 Department of Pathology, University Medical Center Utrecht, Utrecht, The Netherlands; 2 Division of Molecular Pathology, The Netherlands Cancer Institute, Amsterdam, The Netherlands; 3 Vascular Oncology and Metastasis, German Cancer Research Center (DKFZ), Heidelberg, Germany; 4 Vascular Biology and Tumor Angiogenesis, Medical Faculty Mannheim, Heidelberg University, Heidelberg, Germany; 5 Department of Medical Oncology, Cancer Center, UMC Utrecht, Utrecht, The Netherlands

**Keywords:** p120, Mouse model, Breast cancer, Invasive lobular carcinoma

## Abstract

Loss of E-cadherin expression is causal to the development of invasive lobular breast carcinoma (ILC). E-cadherin loss leads to dismantling of the adherens junction and subsequent translocation of p120-catenin (p120) to the cytosol and nucleus. Although p120 is critical for the metastatic potential of ILC through the regulation of Rock-dependent anoikis resistance, it remains unknown whether p120 also contributes to ILC development. Using genetically engineered mouse models with mammary gland-specific inactivation of E-cadherin, p120 and p53, we demonstrate that ILC formation induced by E-cadherin and p53 loss is severely impaired upon concomitant inactivation of p120. Tumors that developed in the triple-knockout mice were mostly basal sarcomatoid carcinomas that displayed overt nuclear atypia and multinucleation. In line with the strong reduction in ILC incidence in triple-knockout mice compared to E-cadherin and p53 double-knockout mice, no functional redundancy of p120 family members was observed in mouse ILC development, as expression and localization of ARVCF, p0071 or δ-catenin was unaltered in ILCs from triple-knockout mice. In conclusion, we show that loss of p120 in the context of the p53-deficient mouse models is dominant over E-cadherin inactivation and its inactivation promotes the development of basal, epithelial-to-mesenchymal-transition (EMT)-type invasive mammary tumors.

**Electronic supplementary material:**

The online version of this article (doi:10.1007/s10911-016-9358-3) contains supplementary material, which is available to authorized users.

## Introduction

Loss of E-cadherin is a driver event in cancer that has been linked to tumor development and progression [1]. In breast cancer, mode and timing of E-cadherin inactivation appears to determine tumor type and etiology [2]. In diffuse gastric cancer and invasive lobular breast cancer (ILC), E-cadherin loss is an early and causative lesion [3–7], while most other tumors show loss of E-cadherin during later stages of disease progression (reviewed in: [8]). Cre-lox based conditional mouse models have demonstrated that mutational inactivation of E-cadherin in the mammary gland is not tolerated, leading to clearance of E-cadherin negative cells [9, 10]. However, in the context of p53 deficiency, E-cadherin loss induces the formation and progression of mouse ILC (mILC), which mimics its human counterpart in phenotype and metastatic dissemination [10, 11].

As an integral part of the adherens junction (AJ), E-cadherin is responsible for homotypic cell-cell connections [12]. E-cadherin stability and turnover is regulated by p120-catenin (p120), an armadillo-repeat containing molecule that binds directly to the E-cadherin juxtamembrane domain at the cell cortex [13, 14]. In ILC cells, loss of E-cadherin results in a translocation of p120 to the cytosol [5, 15, 16], where it controls constitutive activation of autocrine induced RhoA-Rock signaling, which underpins actomyosin-dependent anoikis resistance and subsequent tumor dissemination [17, 18]. In contrast, p120 expression patterns in ductal breast cancers are not related to E-cadherin expression [19]. Localization of p120 can therefore be used to aid differential diagnosis between ductal and lobular breast cancer [16, 20].

Three closely related p120 family members can be found in vertebrates: ARVCF, δ-catenin (CTNND2) and p0071 (PKP4) [21]. Although these family members can also bind and stabilize E-cadherin [22], their redundancy in relation to each other has only been partially addressed. Of note, p120 appears to have evolved together with the non-neural classical cadherins, separately from ARVCF, δ-catenin and p0071, suggesting different functional roles [21].

Although loss of p120 leads to a dissociation of the AJ, conditional loss of p120 in the mouse mammary gland in combination with p53 does not lead to ILC formation but instead induces the formation of high-grade metaplastic-type ductal tumors that metastasize to lungs and lymph nodes [23]. These observations showed that, although the effect of AJ inactivation is the acquisition of tumor invasion and metastasis, the phenotypical outcome of the resulting tumor is determined by the AJ member that is inactivated. To study the contribution of p120 to the development of ILC we introduced a p120 conditional allele [24] into the mILC mouse model [11], and observed that concomitant loss of p120 at the early stages of tumor development largely prevents the formation of mouse ILC (mILC). Our studies indicate that p120 is a crucial factor in ILC etiology.

## Results

### Early Loss of p120 Constrains Formation of ILC in *Wcre;Cdh1*^*F/F*^;*Trp53*^*F/F*^ Female Mice

**Fig. 1:**
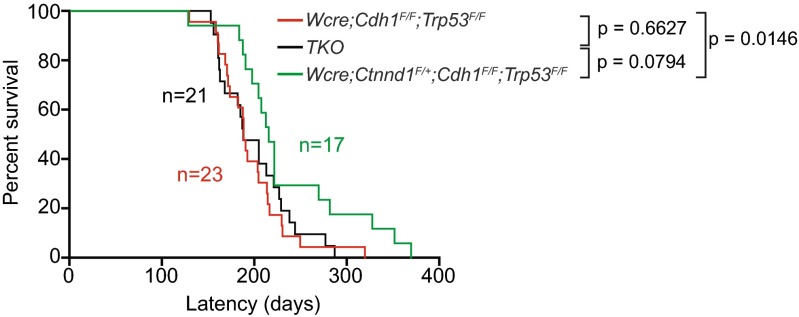
Tumor incidence in Wcre females carrying conditional *Ctnnd1*, *Cdh1* and *Trp53* alleles. Kaplan-Meier tumor-free survival curves are shown for mammary tumors from *Wcre;Cdh1* ^*F/F*^;*Trp53* ^*F/F*^ (*red curve*) TKO (*black curve*) and *Wcre*;*Ctnnd1* ^*F/+*^;*Cdh1* ^*F/F*^;*Trp53* ^*F/F*^ (*green curve*). Mice were sacrificed when tumors reached an average diameter of 10 mm

Using two independent tissue-specific Cre drivers (*K14cre* and *Wcre*) it was previously shown that combined loss of E-cadherin (encoded by *Cdh1*) and p53 (encoded by *Trp53*) in mouse mammary epithelium results in the formation of tumors that resemble human invasive lobular carcinoma (ILC) [10, 11]. To study the contribution of p120 to the development of ILC we introduced a previously generated conditional p120 (*Ctnnd1* ^*F*^) allele [24] into the *Wcre;Cdh1* ^*F/F*^;*Trp53* ^*F/F*^ ILC model [11] to produce *Wcre*;*Ctnnd1* ^*F/+*^;*Cdh1* ^*F/F*^;*Trp53* ^*F/F*^ and *Wcre;Ctnnd1* ^F/F^;*Cdh1* ^*F/F*^;*Trp53* ^*F/F*^ (Triple Knock Out: TKO) female mice that were followed for tumor formation. Although heterozygous inactivation of p120 in the mILC model influenced the median tumor-free survival (T50; 214 versus 178 days, *p* = 0,0146), TKO female mice showed similar T50 values when compared to *Wcre;Cdh1* ^*F/F*^;*Trp53* ^*F/F*^ mice (188 versus 187 days; *p* = 0.6627) (Fig. 1). We also observed no significant T50 differences when comparing *Wcre*;*Ctnnd1* ^*F/+*^;*Cdh1* ^*F/F*^;*Trp53* ^*F/F*^ versus TKO female mice (Fig. 1).

**Fig. 2:**
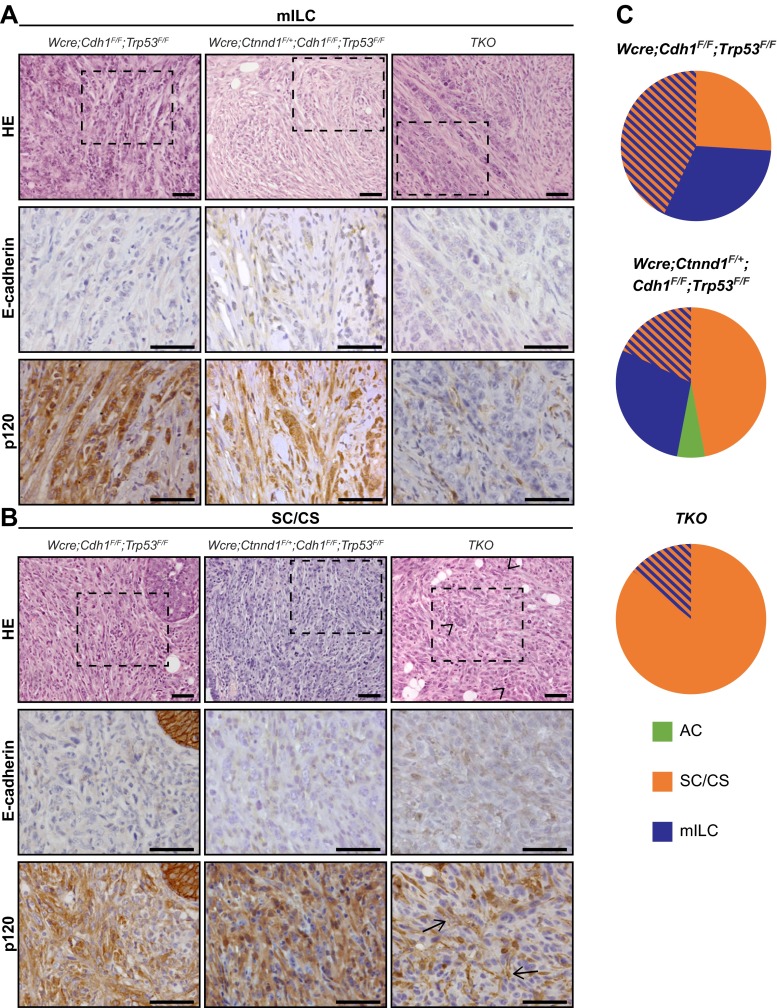
E-cadherin and p120 expression in the mammary tumor spectrum. **a-b** Sections showing representative examples for mouse ILC (mILC) (**a**) and solid carcinoma/carcinosarcoma (SC/CS) (**b**) for the different genotypes studied. Sections were stained for E-cadherin (*middle panels*) and p120 (*bottom panels*). In TKO tumors p120 is only expressed by stromal cells (*arrows*). Nuclear atypia is present in p120 negative tumors (*arrow heads*). The middle and bottom panels are magnifications that correspond to the area indicated in the upper panels. Size bar =25 μm. **c** Distribution of AC (*green*), mILC (*blue*) and SC/CS tumors (*orange*) in mammary glands of *Wcre;Cdh1* ^*F/F*^;*Trp53* ^*F/F*^, *Wcre*;*Ctnnd1* ^*F/+*^;*Cdh1* ^*F/F*^;*Trp53* ^*F/F*^ and TKO female mice

**Fig. 3:**
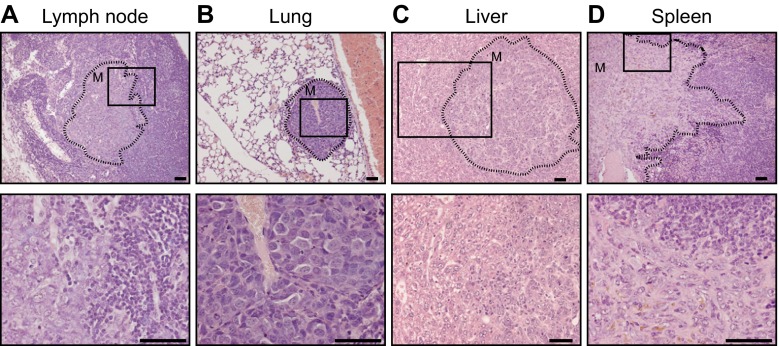
Metastases of mouse tumors from the *TKO* mouse model. Tumor dissemination showing metastasis into the axillary lymph node (**a**), lungs (**b**), liver (**c**) and spleen (**d**). Primary tumors were diagnosed as carcinosarcoma. Dotted lines outline the metastatic area (M). The bottom panels are magnifications that correspond to the area indicated in the top panels. Size bar =25 μm

To explore if p120 controls ILC development we examined tumor histopathology based on H&E staining and diagnosed all primary tumors (Table 1, and Supplementary Table 1). Interestingly, heterozygous deletion of p120 in *Wcre*;*Ctnnd1* ^*F/+*^;*Cdh1* ^*F/F*^;*Trp53* ^*F/F*^ female mice resulted in a marked increase in the incidence of solid ILC compared to *Wcre;Cdh1* ^*F/F*^;*Trp53* ^*F/F*^ control mice (47.1 % versus 4.3 %; *p* = 0.02, Supplementary Table 1 and [11]). Solid-type mILC is a rare ILC subtype characterized by large solid sheets of uniform cells with round nuclei and little stroma, and was only rarely diagnosed in the *Wcre;Cdh1* ^*F/F*^;*Trp53* ^*F/F*^ control cohort (Derksen, 2011). We did not observe statistically significant changes regarding formation of ILC, solid adenocarcinoma (AC) or solid carcinoma/carcinosarcoma (SC/CS) which were characterized by a metaplastic and biphasic histology which comprised mesenchymal elements (Fig. 2) [11, 25]. Furthermore, the percentage of invasive tumors or tumor dissemination (lungs or lymph nodes) did not change upon heterozygous deletion of p120 (Table 1). As with the *Wcre;Cdh1* ^*F/F*^;*Trp53* ^*F/F*^ model we detected occasional tumor dissemination to the abdominal organs, including spleen and liver, in *Wcre*;*Ctnnd1* ^*F/+*^;*Cdh1* ^*F/F*^;*Trp53* ^*F/F*^ and TKO female mice (1 and 2 cases respectively, Supplementary Table 1 and Fig. 3).

**Table 1:** Comparative tumor spectrum and invasiveness

	*Wcre;Cdh1* ^*F/F*^;*Trp53* ^*F/F*^ (n = 23)	*Wcre*;*Ctnnd1* ^*F/+*^;*Cdh1* ^*F/F*^;*Trp53* ^*F/F*^ (n = 17)	p value (vs. *Wcre;Cdh1* ^*F/F*^;*Trp53* ^*F/F*^)	*TKO* (*n* = 21)	p value (vs. *Wcre*;*Cdh1* ^*F/F*^; *Trp53* ^*F/F*^)
Invasive	20 (86 %)	15 (88 %)	*p* = 1.0000	19 (90 %)	*p* = 1.0000
Metastasis	17 (74 %)	7 (41 %)	*p* = 0.0531	10 (48 %)	*p* = 0.1210
AC	0 (0 %)	1 (6 %)	*p* = 0.4250	0 (0 %)	-
SC/CS	16 (70 %)	11 (65 %)	*p* = 1.0000	21 (100 %)	*p* = 0.0094
mILC	17 (74 %)	8 (47 %)	*p* = 0.1074	3 (14 %)	*p* < 0.0001

**Fig. 4:**
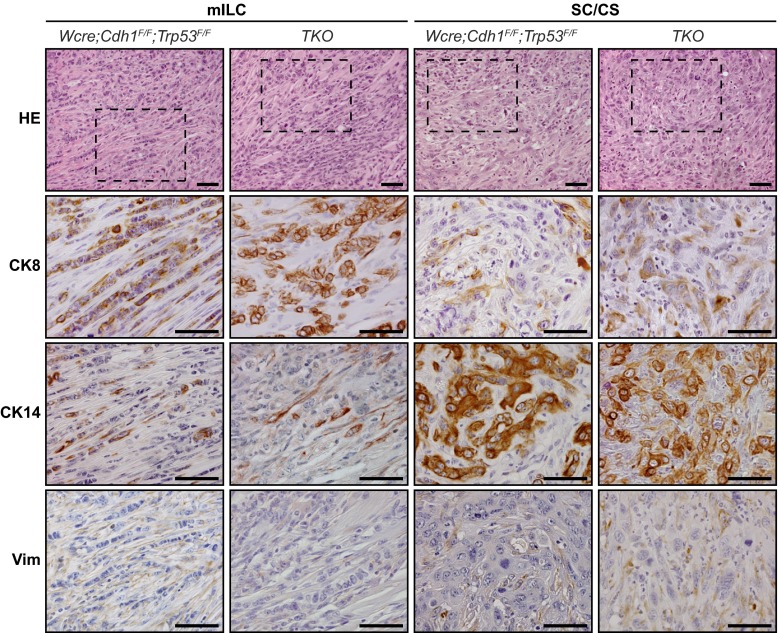
Comparative immunohistochemistry of mammary tumors. Analysis of marker expression in tumor types of *Wcre;Cdh1* ^*F/F*^;*Trp53* ^*F/F*^ and TKO mice. Luminal cells were identified by CK8 expression, while CK14 staining was performed to detect basal cells. Vimentin (Vim) was used as a mesenchymal marker. The bottom panels are magnifications that correspond to the area indicated in the top panels. Size bar =50 μm

Homozygous deletion of p120 in TKO female mice resulted in a tumor spectrum of mainly SC/CS lesions (*p* = 0.0094 compared to *Wcre;Cdh1* ^*F/F*^;*Trp53* ^*F/F*^ mice; Table 1 and Fig. 2) that showed a metaplastic and biphasic histology with overt nuclear atypia and multinucleation. Interestingly, formation of mouse ILC was nearly absent upon homozygous p120 loss (*p* < 0.0001 compared to *Wcre;Cdh1* ^*F/F*^;*Trp53* ^*F/F*^ mice; Table 1). All tumors that developed in the female TKO cohort lacked p120 and E-cadherin expression (Fig. 2 and Supplementary Table 1). In contrast, all tumors from the heterozygous *Wcre*;*Ctnnd1* ^*F/+*^;*Cdh1* ^*F/F*^;*Trp53* ^*F/F*^ mice expressed cytoplasmic and nuclear p120, identical to the expression pattern of p120 in *Wcre;Cdh1* ^*F/F*^;*Trp53* ^*F/F*^ mice (Fig. 2 and Supplementary Table 1). We did not detect overt differences in cytokeratin (CK) and vimentin expression in tumors from the *Wcre;Ctnnd1* ^*F/+*^;*Cdh1* ^*F/F*^;*Trp53* ^*F/F*^ and TKO cohort compared to tumors that had developed in *Wcre;Cdh1* ^*F/F*^;*Trp53* ^*F/F*^;*Trp53* ^*F/F*^ mice (Supplementary Table 1). Overall, tumors showed a mutually exclusive expression pattern of CK8 and CK14, while vimentin was expressed at low levels, mostly in the sarcomatoid/mesenchymal-type tumors (SC/CS) (Fig. 4). None of the tumor types expressed the estrogen or progesterone receptor (ER, PR), in line with previous observations that ER and PR are not expressed in tumors that developed in Wcre; Trp53^F/F^ and *Wcre;Cdh1* ^*F/F*^;*Trp53* ^*F/F*^ female mice [10, 11]. The metastases that formed in *Wcre*;*Ctnnd1* ^*F/+*^;*Cdh1* ^*F/F*^;*Trp53* ^*F/F*^ and TKO displayed marker expression patterns similar to the primary tumors (Supplementary Fig. 1 and Supplementary Table 2). Taken together, our data show that early inactivation of p120 in the context of combined E-cadherin and p53 loss largely prevents formation of mouse ILC, and leads to the formation of high-grade basal mammary tumors that are characterized by a more prominent expression of the basal markers CK14 and vimentin, metaplastic and sarcomatoid histology and strong nuclear atypia.

### p120 Family Members Lack Functional Redundancy during Mammary Carcinoma Development

**Fig. 5:**
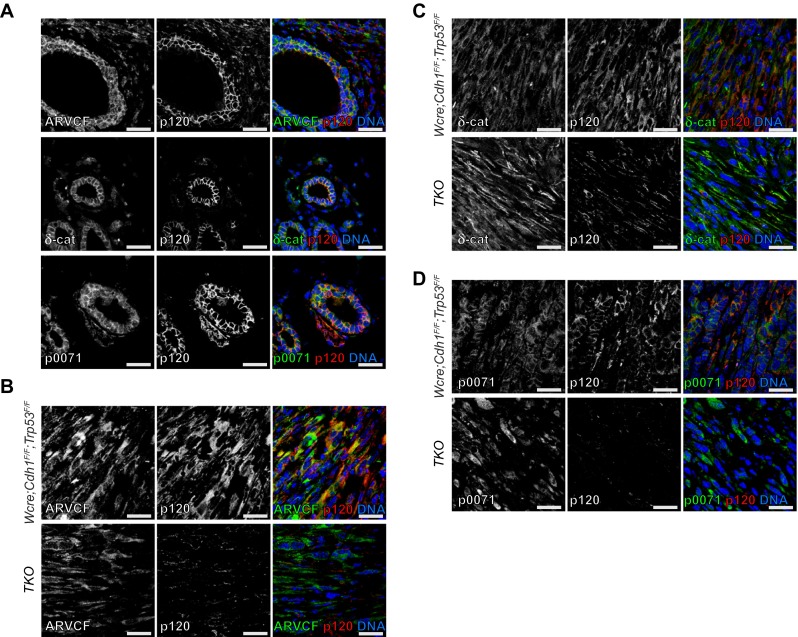
Expression of p120 family members in mILC. **a** p120 family member expression (left panels, green) in the mouse mammary gland. Expression of p120 is depicted in the middle panels (*red*). **b**-**d** Immunofluorescence showing expression of p120 (red) and its family members (green) ARVCF (**b**), δ-catenin (**c**) and p0071 (**d**) in mILCs from *Wcre;Cdh1* ^*F/F*^;*Trp53* ^*F/F*^ (*top panels*) and TKO mice (*bottom panels*). DAPI (*blue*) was used to visualize nuclei. The merged images are shown in the right panels. Size bar =25 μm

Because we diagnosed three p120-negative tumors as mILC in female TKO mice, we wondered whether compensation by one or more p120 family members could have accounted for the formation of these sporadic ILC tumors. Given that ARVCF, δ-catenin and p0071 are structurally related to p120 and share several functions at the membrane and in the cytosol, we investigated the effects of p120 loss on the expression and localization of these family members in mILC. We started by testing specificity of the antibodies by assessing expression in normal mammary epithelial ductal structures using immunofluorescence. Expression of ARVCF, δ-catenin and p0071 in normal ducts showed partial overlap with p120 at the plasma membrane (Fig. 5a). However, we also observed ARVCF, δ-catenin and p0071 expression in the cytoplasm and nucleus (Fig. 5a, left panels).

Next we determined the expression of ARVCF, δ-catenin and p0071 in the different tumor sub-types from TKO mice, which were compared to tumors from *Wcre;Cdh1* ^*F/F*^;*Trp53* ^*F/F*^ mice. In accordance with previous findings we observed cytosolic and nuclear p120 expression in mILC and SC/CS tumors from *Wcre;Cdh1* ^*F/F*^;*Trp53* ^*F/F*^ mice (Fig. 5b-d, top lanes and Supplementary Fig. 2), while in TKO mice p120 expression was only observed in stromal cells (Fig. 5 and Supplementary Fig. 2, bottom lanes). ARVCF, δ-catenin and p0071 showed diffuse localization patterns regardless of tumor type or p120 status (Fig. 5 and Supplementary Fig. 2). Based on these results and the fact that mILC incidence is drastically reduced in TKO tumors, we conclude that ARVCF, p0071 or δ-catenin do not play redundant roles in mammary tumor formation in TKO mice.

## Discussion

Cytoplasmic p120 is a hallmark of lobular breast cancer [5, 15–17]. Here we examined the contribution of p120 to ILC development in mice, and demonstrate that p120 is critical for the development of invasive lobular carcinoma in mice.

Previous data demonstrated that dual inactivation of p120 and p53 in the mouse mammary gland leads to sarcomatoid, epithelial-to-mesenchymal-transition (EMT)-like mammary carcinomas. These p120 negative mammary tumors presented with anaplastic histological features and expression of basal markers such as CK14 and/or vimentin [23]. In this study we have introduced conditional p120 alleles into the WAP-cre driven mouse ILC model [11]. Mouse ILC (like most conditional mouse models of human breast cancer) is an ER negative tumor type [10, 11], and as such models a minority of human ILC that is either ER negative or non-responsive to ER antagonists treatment. Moreover, because mouse ILC appears refractory to cisplatin, docetaxel or doxorubicine (our unpublished results), we use mouse ILC as a model for advanced-stage, metastatic and chemo-refractory human ILC. In contrast, poorly differentiated basal tumors, which we also observe in tumors lacking p120, are usually devoid of ER expression [26]. Although Cre expression in our WAPcre mouse models is mostly restricted to luminal cells, basal ductal cells (CK14^POS^/CK8^NEG^) occasionally also express Cre [11]. Because Cre expression is already evident in virgin *Wcre;Cdh1* ^*F/F*^;*Trp53* ^*F/F*^ female mice, and tumor incidence occurrs independent of parity [11], tumorigenesis in these models is most likely instigated in a mammary progenitor cell type. Because of these data and the fact that most mILC in *Wcre;Cdh1* ^*F/F*^;*Trp53* ^*F/F*^ female mice predominantly expressed CK8, we assume that p120 could play a role in the progression of a luminal-type cancer-initiating cell. Indeed, we show that early dual loss of E-cadherin and p120 almost completely prevents development of mILC. Furthermore, the mILCs that arose in a minority of TKO female mice, displayed biphasic features and consisted mostly of mesenchymal SC/CS tumor cells. Finally, all other tumors that developed in p120 knockout mice were diagnosed as basal-type invasive carcinosarcomas, indicating that loss of p120 predisposes mammary progenitors to a basal lineage commitment and prevents the formation of luminal type ILC.

In spite of this shift towards a basal tumor spectrum we still observed occasional formation of classical mILC lesions in TKO female mice. TKO mILC expressed the luminal CK8 marker, which might be a result of p120 deletion in a luminal-type committed progenitor. We studied expression of ARVCF, p0071 and δ-catenin in TKO mILC lesions, which yielded no indication that p120 loss induced a change in expression levels or localization of these proteins. While this does not formally exclude the possibility of functional redundancy, we conclude that this is not a likely scenario underpinning the occasional formation of ILC in TKO female mice. Also the fact that inactivation of p120 in the context of p53 loss leads to basal-type invasive and metastatic mammary tumors, renders a redundant role for these p120 family members highly unlikely.

**Fig. 6:**
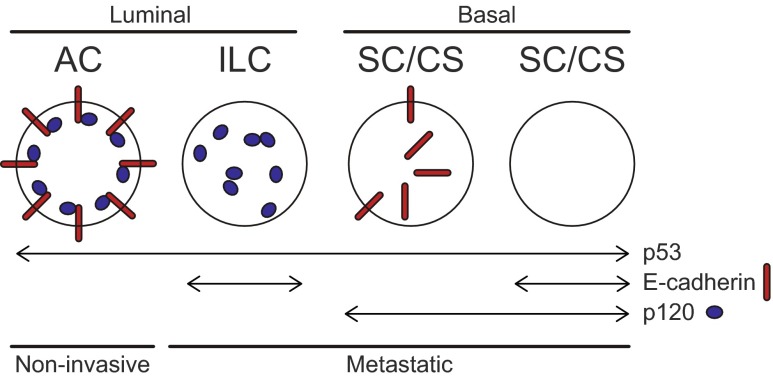
Inactivation of E-cadherin or p120 has divergent consequences on mammary tumor etiology. WAP-cre dependent stochastic loss of p53 predisposes tumor-initiating cells to the development of non-metastatic luminal adenocarcinoma (AC) and basal solid carcinoma/carcinosarcoma (SC/CS) [11]. Inactivation of E-cadherin in this context drives the luminal tumor spectrum towards metastatic invasive lobular cancer (ILC). In contrast, p120 ablation tilts the balance towards formation of basal EMT-type SC/CS. In general, conditional loss of either E-cadherin or p120 results in invasive and metastatic tumors. Inactivation of the adherens junction through p120 loss is dominant over E-cadherin loss in TKO mice, largely preventing ILC development, and driving the formation of EMT-type basal mammary tumors. Arrows indicate mammary-specific Cre-LoxP mediated gene inactivation

Forced dual inactivation of p120 and p53 at the early stages of tumor development might induce two scenarios. First, p120 loss induces an EMT leading to high-grade, basal-type tumors that show a large degree of nuclear atypia and metastasize to lungs and lymph nodes. This process is dominant over E-cadherin loss and its downstream biochemical consequences, preventing ILC formation in triple-knockout mice (Fig. 6). We envisage a second scenario, in which E-cadherin loss triggers cytosolic p120 to provide cues essential for ILC development and progression. Concomitant inactivation would then remove p120 from the ILC-initiating cells and enforce a p120-negative basal breast cancer phenotype. We hypothesize that either scenario can contribute equally to the observed tumor phenotypes.

In human breast cancer, loss of p120 is observed in approximately 15–20 % of invasive ductal carcinomas, with a marked complete loss of p120 expression in metaplastic breast cancer [17, 23, 27–29]. Despite the fact that conditional p120 inactivation in the mouse mammary gland induces invasive mammary carcinomas, human p120-negative breast cancers are mostly devoid of inactivating CTNND1 mutations and do not show silencing through promotor methylation. Moreover, p120 is mostly lost focally in human IDC, indicating that p120 inactivation is a late event in breast cancer. This notion is supported by the fact that (i) p120 loss in the absence of additional oncogenic mutations will negatively impact both luminal and myoepithelial cells of the mammary gland through destabilization of all classical cadherins, and (ii) p120 controls key biological processes such as activation of Rho-dependent actomyosin contractility and distinct transcriptional programs through Kaiso [30–32]. Thus, although p120 loss can propel tumor progression in both cell lineages, this probably only occurs during later stages of human breast cancer progression.

In sum, we show that loss of p120 promotes the development of EMT-type basal invasive mammary tumors. In a p53-deficient context, p120 loss is dominant over E-cadherin inactivation in driving mammary tumorigenesis, thus largely preventing the formation of ILC. Conversely, in the context of early mutational E-cadherin activation, p120 will take center stage to unveil its oncogenic role to drive anchorage-independence and metastatic ILC.

## Materials and Methods

### Generation and Genotyping of *Wcre*;*Ctnnd1*^*F/+*^;*Cdh1*^*F/F*^;*Trp53*^*F/F*^ and TKO Mice

Mammary-specific p120 knockout mice were generated by crossing the conditional *Ctnnd1* ^F^-allele [24] onto the *Wcre;Cdh1* ^*F/F*^;*Trp53* ^*F/F*^ mice [11]. Genotyping was done as described previously [10, 11, 24]. Mice were euthanized when tumors reached a diameter of 10 mm. Date of sacrifice was used for the tumor-free survival analyses. Histology of the primary tumor was used as diagnosis while full autopsies were performed to detect additional tumors and metastases. All animal experiments were approved by the Animal Ethics Committee (DEC) of the Netherlands Cancer Institute (DEC-A: 09,014, DEC-B: 2/6, work protocol 3620.1939). *Wcre;Cdh1* ^*F/F*^;*Trp53* ^*F/F*^ mouse data were described previously [11].

### Antibodies

Primary antibodies used include mouse anti-p120-catenin (1:500, BD Biosciences 610134), mouse anti-E-cadherin (1:200, BD Biosciences 610182) rat anti-CK8 (1:125, Developmental Studies Hybridoma Bank, Troma-1), rabbit anti-CK14 (1:10.000, Covance, PRB-155P), guinea pig anti-vimentin (1:400, Fitzgerald, 20R-VP004), rabbit anti-δ-catenin (EMD-millipore, 07–259), guinea pig anti-ARVCF (1:100, previously used in [33]), guinea pig anti-p0071 (1:100, previously used in [33]).

Secondary antibodies used were rabbit anti-guinea pig (DAKO, p0141), HRP conjugated rabbit anti-rat (DAKO p0450), poly HRP anti-rabbit (Immunologic, DPVR500HRP), poly HRP anti-mouse/rabbit/rat (Immunologic, DPVO500HRP), Alexa568 conjugated anti-mouse (1:500, Invitrogen, A11031) and Alexa488 conjugated anti-rabbit (1:500, Invitrogen, A11034).

### Immunohistochemistry and Immunofluorescence

Tissues were fixed in 4 % formaldehyde for 24 h. After dehydration, tissues were embedded in paraffin and cut into 4 mm sections. Slides were rehydrated and endogenous peroxidase was blocked in 1.5 % H_2_O_2_ containing buffer. Depending on the primary antibody used, antigen retrieval was performed either by proteinase K (DAKO) treatment for 5 min (for vimentin and CK8) or by boiling of the slides in 10 mM citrate buffer (pH 6.0) for 20 min (for E-cadherin and p120). For CK14 no additional procedure for antigen retrieval was performed. Primary antibody incubation took place overnight at 4 °C, followed by staining with HRP-conjugated secondary antibodies for 30 min. The substrate was developed using diaminobenzidine (DAB) followed by hematoxylin staining. Finally, sections were dehydrated and mounted with pertex for microscopic examination. For immunofluorescence, sections were incubated with Alexa conjugated secondary antibodies for 2 hours followed by DAPI incubation to stain for DNA. The slides were mounted using Vectashield fluorescence (Vector Labs) and images were collected by confocal laser microscopy using a Zeiss LSM510 Meta.

### Statistical Analysis

Statistics were calculated using Graphpad Prism 5. For survival analysis, the Log-Rank test was used. For analysis of growth patterns, metastasis and histological types, Fisher’s exact test was used. *P* values <0.05 were considered statistically significant.

## Electronic supplementary material

Fig. S1 Comparative immunohistochemistry of metastasis in TKO mice. Analysis of marker expression in metastasis of TKO mice in an axillary lymph node (**a**), lung (**b**), liver (**c**) and spleen (**d**). Primary tumors were diagnosed as carcinosarcoma. M marks the metastatic tissue. Size bar =50 μm. (PDF 2492 kb)

Fig. S2 Expression of p120 family members in SC/CS tumors from Wcre;Cdh1^F/F^;Trp53^F/F^ and TKO mice. a-c. Expression of ARVCF (**a**, green), δ-catenin (**b**, green) p0071 (**c**, green) and p120 (**a-c**, red) in SC/CS tumors from Wcre;Cdh1^F/F^;Trp53^F/F^ (top panels) and TKO mice (bottom panels). DAPI (blue) was used to visualize nuclei. The merged images are shown in the right panels. Size bar =25 μm (PDF 1411 kb)

Table S1 (PDF 148 kb)

Table S2 (PDF 117 kb)
