# Supplementary material for: p120-Catenin Is Critical for the Development of Invasive Lobular Carcinoma in Mice
Source: J Mammary Gland Biol Neoplasia. 2016 Jul 13;21(3):81–8. doi: 10.1007/s10911-016-9358-3 (PMC5159444; doi:10.1007/s10911-016-9358-3)
Supplement: Supplementary file 1 — Comparative immunohistochemistry of metastasis in TKO mice. Analysis of marker expression in metastasis of TKO mice in an axillary lymph node (a), lung (b), liver (c) and spleen (d). Primary tumors were diagnosed as carcinosarcoma. M marks the metastatic tissue. Size bar =50 μm. (PDF 2492 kb) [file 10911_2016_9358_MOESM1_ESM.pdf]

Supplementary Figure 1

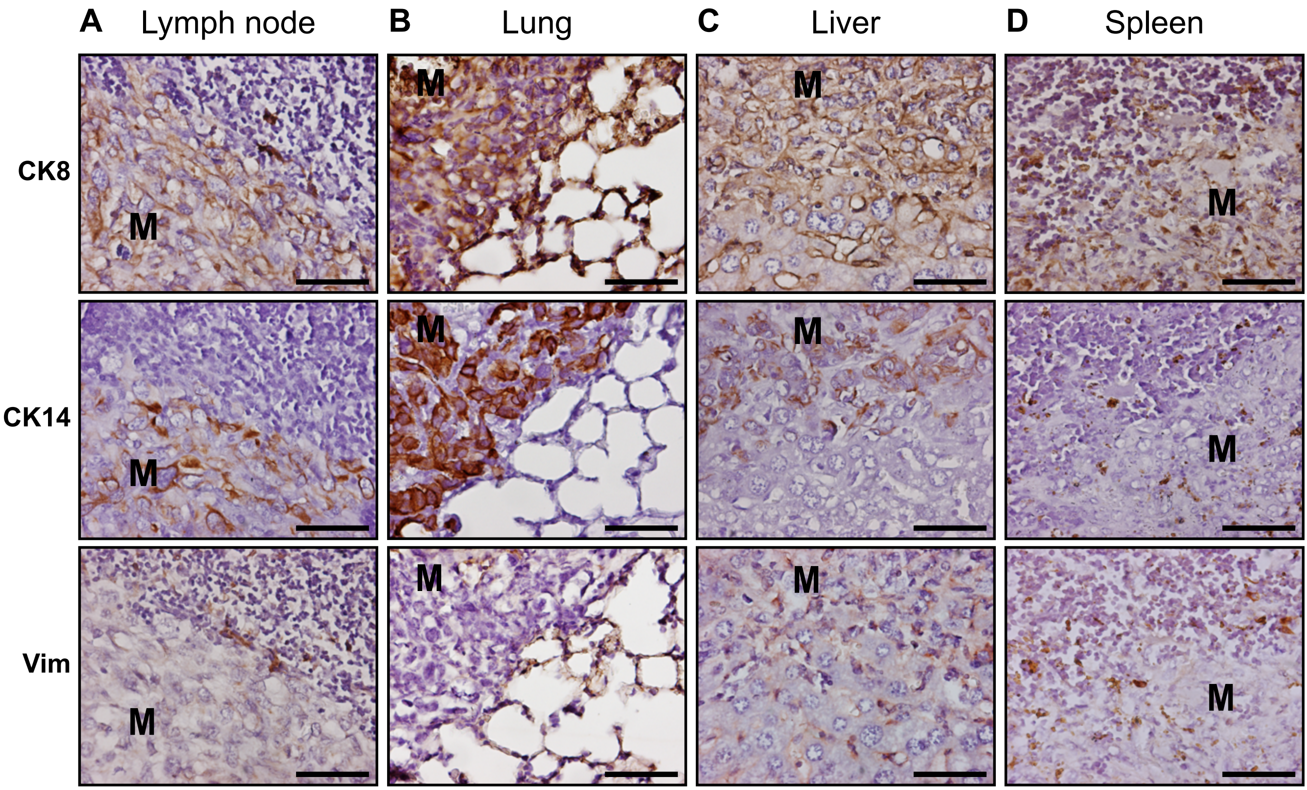

Tenhagen *et al. p120-catenin is critical for the development of invasive lobular carcinoma in mice*

Journal of mammary gland biology and neoplasia. Corresponding author: P.W.B. Derksen, UMC Utrecht, pderksen@umcutrecht.nl
