# Supplementary figures and images for: p120-Catenin Is Critical for the Development of Invasive Lobular Carcinoma in Mice
Source: J Mammary Gland Biol Neoplasia. 2016 Jul 13;21(3):81–8. doi: 10.1007/s10911-016-9358-3 (PMC5159444; doi:10.1007/s10911-016-9358-3)

# Supplementary Figure 2

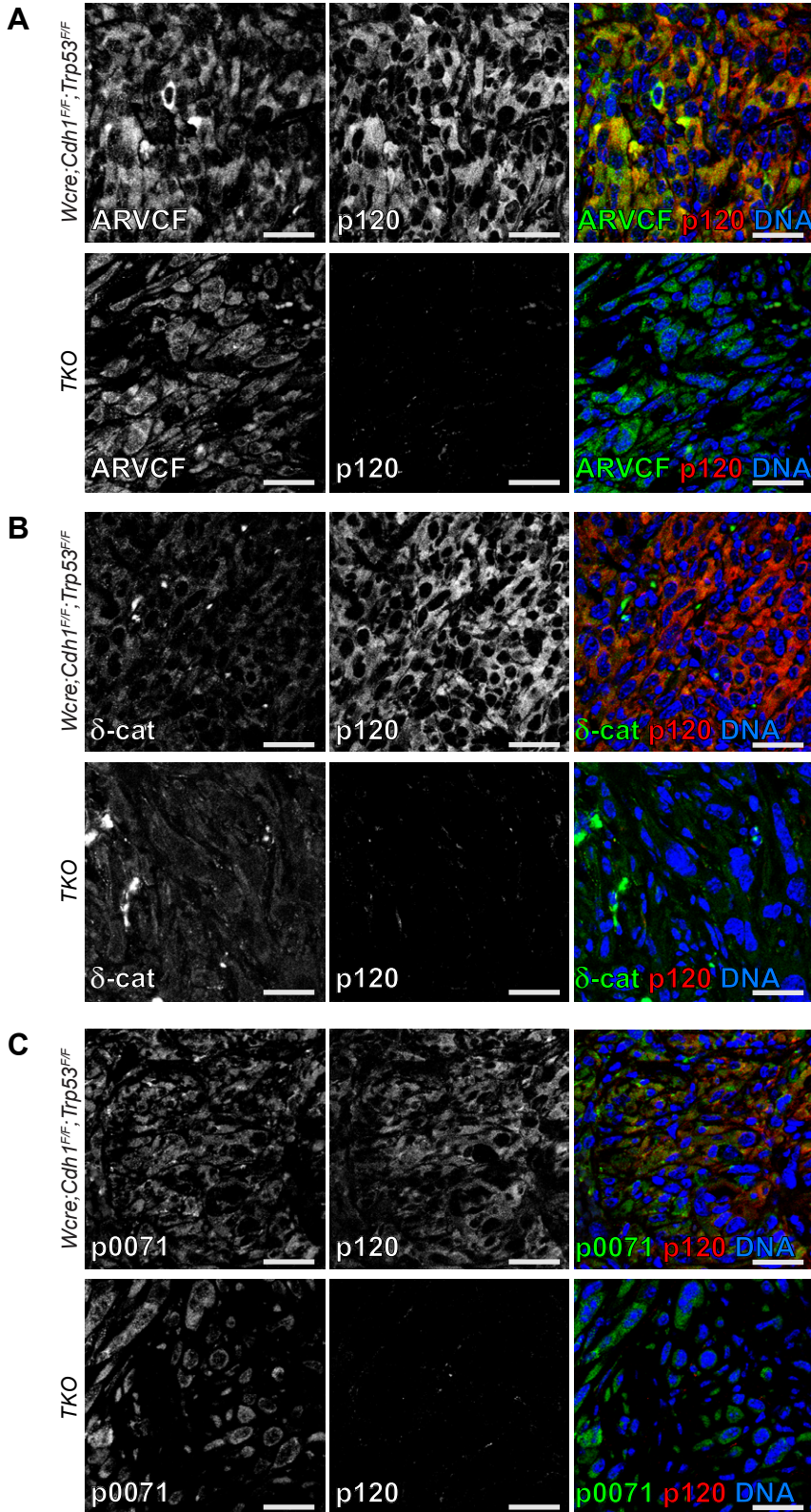

Supplement: Supplementary file 2 — Expression of p120 family members in SC/CS tumors from Wcre;Cdh1F/F;Trp53F/F and TKO mice. a-c. Expression of ARVCF (a, green), δ-catenin (b, green) p0071 (c, green) and p120 (a-c, red) in SC/CS tumors from Wcre;Cdh1F/F;Trp53F/F (top panels) and TKO mice (bottom panels). DAPI (blue) was used to visualize nuclei. The merged images are shown in the right panels. Size bar =25 μm (PDF 1411 kb) [file 10911_2016_9358_MOESM2_ESM.pdf]
