# Supplementary material for: p120-Catenin Is Critical for the Development of Invasive Lobular Carcinoma in Mice
Source: J Mammary Gland Biol Neoplasia. 2016 Jul 13;21(3):81–8. doi: 10.1007/s10911-016-9358-3 (PMC5159444; doi:10.1007/s10911-016-9358-3)
Supplement: Supplementary file 3 — (PDF 148 kb) [file 10911_2016_9358_MOESM3_ESM.pdf]

Supplementary table 1: Comparative histopathology

	Mouse	Age (days)	Primary tumor(s)	p120	Ecad	CK8	CK14	Vim	metastasis
WCre;Ctnnd1F/+;Cdh1F/F;Trp53F/F	09DER004	127	mILC (solid)	+	-	±	±	+	no
	10DER011	182	mILC (solid)	+	-	-	+	±	lungs
	10SJK252	186	AC	+	-	ND	+	-	no
	09DER011	189	SC/CS	+	-	±	±	±	no
	10SJK254	196	CS<br>CS	ND<br>+	ND<br>-	ND<br>ND	ND<br>++	ND<br>-	axillary lymph node
	09DER005	203	CS	+	-	-	±	+	no
	10SJK262	206	mILC (solid)	+	-	+	+	±	no
	09SJK136	211	mILC (solid)	+	-	+	+	+	axillary lymph node
	10SJK214	214	CS and mILC (solid)	+	-	-	+	-	no
	09DER007	220	SC/CS	-	-	±	±	±	no
	10DER009	220	mILC (solid)	+	-	++	+	±	lungs
	10DER016	220	CS	+	-	±	+	-	no
	10DER021	268	CS	+	-	±	±	-	abdominal cavity
	10DER007	280	CS and mILC<br>mILC (solid)	+<br>+	-<br>-	+<br>++	±<br>ND	±<br>-	renal, lumbar, caudal lymph nodes
	11SJK032	326	CS and mILC<br>mILC (classic and solid)	ND<br>+	ND<br>-	ND<br>-	ND<br>+	ND<br>-	lung
	10SJK236	350	CS	+	-	±	±	±	no
	10SJK196	368	SC/CS	ND	ND	ND	ND	ND	no
TKO	10DER008	153	CS	-	-	++	+	±	axillary lymph node, lung
	09DER018	156	SC	-	-	-	+++	±	no
	09DER006	161	CS + mILC	-	-	+	+	+*	axillary lymph node, lung
	10DER004	161	SC/CS	-	-	±	±	±	abdominal cavity
	09DER001	162	SC	-	-	±	±	+	no
	09DER019	163	SC	-	-	±	++	+	no
	10DER006	168	CS	-	-	+	++	-	no
	09DER012	182	SC	-	-	+	±	+	axillary lymph node, lung
	09DER008	185	SC	-	-	-	±	+	no
	10DER002	187	SC	-	-	-	+	+	no
	10SJK041	188	CS	-	-	-	±	±	no
	10DER010	205	CS	-	-	+	+	-	no
	10DER015	205	CS	-	-	±	±	-	no
	09DER013	213	CS	-	-	±	++	-	axillary lymph node
	09DER003	221	CS	-	-	±	+	+	lumbal lymph node
	09DER010	227	SC<br>SC + mILC	-<br>-	-<br>-	+<br>+++	+++<br>+	-<br>-	axillary lymph node
	10DER013	229	CS	-	-	+	+	±	axillary lymph node, spleen, liver
	09DER016	238	SC + mILC<br>CS	-<br>-	-<br>-	++<br>+	+<br>±	-<br>±	axillary lymph node
	09DER017	244	SC	-	-	-	±	±	axillary lymph node
	10DER014	277	CS	-	-	±	-	-	no
	10SJK132	287	SC/CS	-	-	±	-	+	no

- = no staining, ±= focal expression in less than 10% of the tumor cells, + = expression ranging between 10% and 40% positive tumor cells, ++ = 40% - 80% positive tumor cells, +++ = > 80% positive tumor cells, * mILC Vim -
