# Supplementary material for: p120-Catenin Is Critical for the Development of Invasive Lobular Carcinoma in Mice
Source: J Mammary Gland Biol Neoplasia. 2016 Jul 13;21(3):81–8. doi: 10.1007/s10911-016-9358-3 (PMC5159444; doi:10.1007/s10911-016-9358-3)
Supplement: Supplementary file 4 — (PDF 117 kb) [file 10911_2016_9358_MOESM4_ESM.pdf]

Supplementary table 2: Comparative histopathology of metastasis

	Mouse	metastasis	CK8	CK14	Vim
WCre;Ctnnd1F/+; Cdh1F/F;Trp53F/F	10DER011	lungs	+	-	-
	10SJK254	axillary lymph node	+	±	-
	09SJK136	axillary lymph node	+	-	+
	10DER009	lungs	+	-	-
	10DER021	abdominal cavity	-	-	-
	10DER007	distant lymph nodes	+	-	-
	11SJK032	lungs	+	-	±
TKO	10DER008	axillary lymph node	+	-	-
		lung	±	+	-
	09DER006	axillary lymph node	+	+	-
		lung	+	+	-
	10DER004	abdominal cavity	+	±	-
	09DER012	lungs	+	+	-
	09DER013	axillary lymph node	+	+	-
	09DER003	lumbal lymph node	+	+	±
	09DER010	axillary lymph node	+	-	-
	10DER013	axillary lymph node	+	-	-
		spleen	+	-	-
		liver	+	+	±
	09DER016	axillary lymph node	+	-	-
	09DER017	axillary lymph node	+	-	+

- = no staining, ±= focal expression in less than 10% of the tumor cells, + = expression in at least 10% of all tumor cells
